# Changing University Students’ Habit Strength Towards Alcohol Consumption Using Affectively and Cognitively Framed Messages

**DOI:** 10.3390/bs15121637

**Published:** 2025-11-28

**Authors:** Benjamin Morris, Tom St Quinton, Mark Conner

**Affiliations:** 1 School of Social Science, Leeds Trinity University, Leeds LS18 5HD, UK; 2 School of Psychology, Leeds Beckett University, Leeds LS2 3AX, UK; t.st-quinton@leedsbeckett.ac.uk; 3 School of Psychology, University of Leeds, Leeds LS2 9JT, UK; m.t.conner@leeds.ac.uk

**Keywords:** affect, habit strength, messaging, alcohol consumption, higher education, health promotion

## Abstract

Excessive alcohol consumption (EAC), defined as consuming more than 14 units per week, in the university population represents a significant health risk to students. Objective: The present research tested the impact of different attitude-salient messages to reduce the strength of habits towards consuming alcohol. Methods: Seven hundred and fifty-five university students were randomised to one of five conditions that varied in the content of attitude salience: short-term affective, short-term cognitive, long-term affective, long-term cognitive, and no message control. The habitual strength of participants’ alcohol consumption was measured at baseline and at follow-up using the Self-reported Habit Index. Results: ANCOVA controlling for the baseline assessed potential gender differences and several significant interactions were identified, demonstrating that the affective distal message reduced habitual strength towards EAC to a greater extent for men low in Need for Cognition (NfC; 95% CI [0.48, 2.12]), whereas, for women low in NfC, the affective proximal message was most effective. For both men and women high in NfC, the cognitive message was shown to be more effective at reducing the strength of habit towards EAC. Conclusion: The findings point to the value of distinguishing between health messages in terms of several factors, including affective and cognitive outcomes, the temporal nature of these outcomes (short-term or long-term), and gender. This has important ramifications for providing information to modify behaviour.

## 1. Introduction

The present work investigates psychological factors influencing excessive alcohol consumption (EAC), defined as consuming more than 14 units of alcohol per week, among university students, with a focus on how different types of health messages influence habit strength towards alcohol consumption. The study draws upon dual-process theories of behaviour, distinguishing between impulsive, habitual responses and deliberate, reflective reasoning. It examines how emotional (affective) and logical (cognitive) information can influence risk perception and decision-making, especially when framed around either short-term or long-term consequences.

### 1.1. Alcohol Consumption in the University Population

EAC is defined as consuming more than 14 units of alcohol per week (Drinkaware, 2025; NHS, 2022). It is a leading cause of disease (World Health Organization [WHO], 2018) and can lead to negative health consequences such as cancer (Rehm et al., 2019), depression (Cheng et al., 2016; McConaha et al., 2024), and increased mortality (Burton & Sheron, 2018). Although alcohol intake is a significant concern across different populations for many of the above reasons, it is a particular concern for students attending university (Cameron et al., 2015; Davoren et al., 2016; Fenton et al., 2023; Riordan & Carey, 2019). Indeed, research has shown that students in the UK consume considerably more alcohol than non-students (Carter et al., 2010; Kypri et al., 2005; Messina et al., 2021), and consumption specifically increases when students begin university (Bewick et al., 2008; Gambles et al., 2021; Turrisi et al., 2006). Whilst levels are reported to fluctuate across the typical three-year period of study, significant peak times for excessive alcohol consumption appear in the first year and towards the end of the third year (Bennasar-Veny et al., 2020; Bewick et al., 2008; Brown & Murphy, 2020). This more broadly represents a culture of UK drinking amongst university students, which can include excessive pre-drinking prior to socialising (Santos et al., 2022), leading to an increased odds of experiencing a range of harms, with drinking characterised as being important for securing new friendships (Gambles et al., 2021) and managing stress (Conroy & Nicholls, 2022). As such, university students are at a particular risk for alcohol-related harm due to a consistent tendency to drink higher levels than recommended (Dantzer et al., 2006; Herrero-Montes et al., 2022). EAC whilst at university can have additional negative consequences such as a reduction in academic performance (Paul et al., 2024; Tembo et al., 2017), increased illness susceptibility (Barnett et al., 2014), and participation in other risky behaviours (Jones et al., 2006; Bartholomew et al., 2021). It can also contribute towards harmful drinking habits in later life (Sjödin et al., 2024; Stickley et al., 2013).

### 1.2. Elaboration Likelihood Model

The Elaboration Likelihood Model (ELM; Petty & Cacioppo, 1986) is a theory of persuasion that explains how attitudes are formed and changed through two different routes, namely the central route and the peripheral route. The central route involves careful and thoughtful processing of a message’s arguments, leading to more durable attitude changes, and is used when a person is motivated and can think deeply. The peripheral route involves a less cognitive approach, where people are influenced by surface-level cues like a speaker’s attractiveness or emotional appeal, and is used when motivation or ability is low. This approach has been shown to be useful when examining alcohol consumption in college students previously (Glassman et al., 2018; Petty et al., 2009).

### 1.3. Need for Cognition

A factor implicated in the present work is the extent to which any effects of an affective-based or cognitive-based message is moderated by the Need for Cognition (NfC). NfC is the disposition for individuals to be receptive to cognitive information. Haddock et al. (2008) showed NfC to moderate the impact of affective and cognitive messages in attitude change such that the messages led to more positive attitudes, a higher message receptivity, and an improved recognition for individuals high in NfC. Similarly, Conner et al. (2011) demonstrated how individuals low in NfC were more influenced by an affective message, compared with a cognitive message, to increase levels of exercise. The potential for interactions between cognitive alcohol expectancies and NfC have been established for some time now (Hittner, 2004; Ingendahl et al., 2021). Capone and Wood (2009) demonstrated how a brief motivational intervention was found to have a stronger effect on drinking behaviour over time for those high in NfC. This speaks to the complexities in emotional regulation and the moderating effects of NfC inherent within individuals who engage in EAC, especially when emotion is being targeted (Le Berre, 2019). Presently, it is predicted that NfC will influence the efficacy of different messages on self-reported habit strength towards alcohol consumption.

### 1.4. Habit

Habit is conceptualised to have several characteristics that make it pertinent to understanding and modifying health behaviour. Dual-process models propose two parallel processing systems (Strack & Deutsch, 2004; Hofmann et al., 2008). Firstly, habit is depicted on an impulsive pathway, such that perceptions of cues activate context–behaviour relationships, prompting behaviour rapidly and with minimal consideration. Secondly, reasoned cognitions (e.g., intentions) are represented on a reflective pathway, where a cue initiates a rule-based deliberation that directs behaviour. The present work emphasises the importance of this second pathway. Accurate risk appraisal is dependent upon the availability of relevant information coupled with effectively using that information to form a judgement (Fischhoff et al., 1978). The comprehension of risk is thus inherently captured by the compelling nature of the health material, such that a health message becomes relevant, in the context of changing behaviour, as a function of its ability to elicit a desired response (Peters, 2006; Rocklage & Luttrell, 2021).

### 1.5. Habit–Emotion Relationship

Emotions offer humans a rich source of information to better understand the relationship between themselves and their world. In doing so, they play a key adaptive role in survival (Gu et al., 2023; Heydari et al., 2021). Presenting information that focuses upon the affective compared with cognitive consequences of a behaviour has been shown to be more effective at instigating change in some instances (e.g., Conner et al., 2011; Morris et al., 2015) and leads to a deeper consideration of information, leading some individuals to engage in personal rather than political or ideological reasoning (Schwarz, 2010; Feldman & Hart, 2018; Bolsen & Palm, 2019). Eliciting affective responses, as well as highlighting the anticipatory affective consequences of a behaviour, has been shown to be influential in a number of common health behaviours such as alcohol consumption (AC), with more affective imagery eliciting increased cravings for smoking (Tiffany & Drobes, 1990), affective ambivalence predicting the likelihood to hold positive attitudes towards organ donation (van den Berg et al., 2005), and conversely negative affective cueing being shown to be a primer for increased AC (Conner et al., 2020; Zack, 2006). Indeed, this may explain why a large proportion of students are said to consume excessive levels of alcohol to manage negative experiences such as anxiety and work stress (A. Williams & Clark, 1998; Frone & Bamberger, 2024), the same mechanism being used in a deleterious direction.

It may be argued that affective-based messages more readily convey this more evocative information through the targeting of such emotional responses (Zeelenberg & Beattie, 1997; see Tannenbaum et al., 2015; Di Plinio et al., 2025, for reviews). Presenting health information in the form of an affective-based message may be predicted to more readily have a health promoting effect upon behaviour (Tiedens & Linton, 2001; Caruso & Shafir, 2006; van Dijk & Zeelenberg, 2006; Mai et al., 2024) than a more typically used cognitive-based message. This comparison was tested in the current study.

### 1.6. Temporal Salience of Outcomes

The expected utility model (Edwards, 1954) acknowledges the role of time in influencing the choices we make; for example, individuals have been found to attribute greater value to short-term rewards compared to later rewards, even in cases when they have the same utility (Hsee & Rottenstreich, 2004). Indeed, where long-term consequences have a greater utility, but are still waylaid in favour of short-term gains, the greater long-term rewards are said to be discounted (Petry, 2001; Seaman et al., 2022). Indeed, short-term and long-term outcomes do not appear to compete on a level playing field when referring to decision-making (Hall & Fong, 2003; Wang et al., 2023). Becker and Murphy (1988) have explained addictive behaviours in terms of this temporal pay-off between short-term and long-term consequences. They state that excessive impulsive behaviour is simply a process of over discounting the consequences of future behaviour such that short-term benefits become more appealing (Glimcher, 2022; Acuff et al., 2024). Thus, these individuals are said to be simply maximising their expected utilities to gain the most immediate gains.

Keough et al. (1999) demonstrated that individuals reporting substance abuse also scored highly on present time perspective (PTP), with future time perspective (FTP) also negatively correlating with reported substance abuse. The consumption of alcohol has been shown to induce myopia in individuals such that they attend to the most immediate stimulus in their environment (Sevincer et al., 2012) and can also significantly impair their adherence to personal norms and standards (Stepanova et al., 2018). Finally, associations with alcohol have been shown to cue aggressive behaviour even in the absence of alcohol consumption (Bartholow & Heinz, 2006). This variance in time perspective can be viewed therefore as an important variable potentially capable of modifying the significance of information, at least on an individual basis (Maddux et al., 1995; Carstensen et al., 1999). The distinction between risks that are close (proximal) compared with risks that are distant (distal) in time has been posited as explaining why certain individuals are prepared to waylay hazards associated with “maladaptive” behavioural patterns. This is supported by the fact that a significant proportion of the western populace continue to drink excessively, despite the well-known health-harming consequences of doing so. As a result, behaviours with long-term health risks can be seen as possessing short-term benefits that, at least in the short-term, make the long-term risks less concerning. In terms of guiding behaviour, time focus can be conceptualised as an individual difference variable explaining why some people (specifically those who focus more on long-term risks) apparently behave “more rationally” in light of potential dangers, whereas others would seem to ignore these same dangers (Hall & Fong, 2007; Epper & Fehr-Duda, 2024).

Whilst nearly all behaviours will have both short- and long-term consequences, the “temporal salience” of these consequences, and the risks appraised, would seem to be directive in decision-making (Evans et al., 2019; McAlpine & Mullan, 2022; Boucher et al., 2025). Strong hedonic, short-term gains may negate long-term risks, whereas strong long-term benefits may be distorted by less compelling short-term cues, and this is supported in the literature (Loewenstein & Thaler, 1989; Loewenstein & Elster, 1992; Ditto et al., 2006; Strathman et al., 1994; P. G. Zimbardo et al., 1997; P. Zimbardo & Boyd, 2008; Mollen et al., 2017). Providing individuals with information on the relevant short-term or long-term consequences of certain behaviours may be sufficient to motivate individuals to modify their behaviour (Ainslie & Haendel, 1983; Shiota, 2021) or to a certain degree engage in less temporal discounting of long-term rewards, as such making these long-term options more amenable.

### 1.7. Health Messages

Providing information to individuals has long represented a dominant approach to behaviour modification for the general population (Sallis et al., 1999; Crawshaw et al., 2022; van Valkengoed et al., 2022). These approaches assume that providing individuals with information regarding the potential hazards of a health-harming activity will be sufficient to modify behaviour (Blalock et al., 1990). However, this is not always supported (e.g., A. Williams & Clark, 1998; Nutbeam, 2000; Corvera-Tindel et al., 2004).

One mechanism by which health information can affect intentions to engage in a behaviour is through its effect on outcome expectancies. Outcome expectancies are subjective probabilities that a specific action will result in a specific outcome. They are conceptually derived from the work of Bandura (1986) and Rotter (1966). Shell (2023) has stated that the expectation of outcome is strongly associated with prior experiences. Vicarious learning plays an increasing role for outcome expectancies where the behaviour and/or outcome is novel (Schunk & DiBenedetto, 2020). Shell and Flowerday (2019) acknowledge that most of our expectancies derive from subjective experiences; as such, one might expect there to be inconsistencies between the deliverer and receiver of the communication in the immediate salience of consequences.

Several potential factors may explain the varying success of providing information to individuals under the auspices of modifying behaviour. Specifically, affective messages (AMs) that are deemed to elicit more actionable outcome expectancies (Feather, 2021; Finucane et al., 2000; Rottenstreich & Hsee, 2001) can be compared with cognitive messages (CMs) that may be predicted to elicit these outcome expectancies less. Strong emotional reactions, more readily facilitated via affective-based material, can often take less time to effect change and can be viewed as shortcuts to decision-making (Benthin et al., 1995; Eberhard, 2023; MacGregor et al., 2000; Slovic et al., 2002), whereas cognitive responses that necessarily require comparably longer deliberation are more amenable to long-term experiences. Based upon this assessment, messages that are framed in terms of affective consequences (e.g., the emotional consequences of behaviour) may have a greater positive influence in the short-term. Whilst messages framed in terms of cognitive consequences (e.g., medical information presented as data) may have greater “weight” over a longer-term, if sufficiently attended to by the individual.

Since risks appraised in terms of their affective and cognitive triggers may be described as typically functioning on different time frames (Lerner et al., 2024; B. R. Williams et al., 2005), it may be valuable to assess whether a focus on short-term or long-term consequences in a message influences decision-making and subsequent behaviour. Since affective information can be viewed as usually residing in an immediate domain of experience it may be hypothesised that affective-based information operates most efficiently when framed in terms of short-term consequences, whereas the opposite may be predicted for cognitive-based information (i.e., works better when focused in terms of longer-term outcomes).

### 1.8. Gender

In what was the first systematic review of emotional processes in relation to binge drinking, Lannoy et al. (2021) identified several emotional components to excessive alcohol consumption, including a heightened negative state (depression and anxiety) and a diminished emotional response to the effects of alcohol. Sawyer et al. (2019) have demonstrated neurological differences between men and women in how they use alcohol to regulate emotions. This suggests a possible difference in aetiology and maintenance of EAC by gender. Studies of subjective emotion experience identify that women report more heightened sadness (Brebner, 2003; Fischer et al., 2004) and anxiety (Feingold, 1994; Ollendick et al., 1995) than men. Dijkstra et al. (2001) indicate that predictors of EAC have been found to differ for men versus women, with Chaplin et al. (2008) elaborating a pathway which states a gender difference in stress response, with women experiencing heightened sadness and anxiety and men showing greater reward motivation behaviour. This has clear implications for a gender-related vulnerability for some alcohol use disorders. This pattern is shared in some other health-related behaviours (Alsharawy et al., 2021).

There are also well documented gender differences in alcohol consumption habits (Goh et al., 2024; Kezer et al., 2021). Nolen-Hoeksema (2004) emphasises the large and consistent gender differences in the consequences of drinking alcohol, with women suffering the serious negative consequences of alcohol consumption earlier and to a greater degree than men. They elaborate that the short-term consequences may discourage most women from EAC, and the distal consequences may create selection pressures against EAC, resulting in lower consumption rates relative to men. In some cases, gender differences can be explained away in terms of empathic accuracy and situational factors that influence the stereotyped expected gender difference (Löffler & Greitemeyer, 2023). In any case, one might expect to identify a gender difference in the efficacy of health messages focusing on affective expectancies of EAC.

### 1.9. Hypothesis

The paper presents a study comparing the effects of a message targeting affect with one targeting cognition and a no message control in changing habit strength. Messages contained information (affective, cognitive) that described the consequences of engaging in AC. This information was then framed further in terms of either the proximal (short-term) or distal (long-term) consequences. Measures of gender and NfC were also assessed as potential factors in the efficacy of the messages. Specifically, the present study seeks to test two hypotheses.

**Hypothesis** **1.** *It is predicted that there will be a difference in the efficacy of a message on changing the habit strength to engage in EAC.*

**Hypothesis** **2.** *It is predicted that the strength of habits towards EAC will be moderated by NfC.*

**Hypothesis** **3.** *It is predicted that there will be a gender difference in message efficacy to modify the strength of habits towards engaging in EAC.*

## 2. Method

### 2.1. Design

The present study adopted a repeated measures design with data collected at two time points spaced seven days apart. The study included five conditions varying by message type (affective versus cognitive) and temporal salience (proximal versus distal), plus a no message control. All participants received a generic message first, and, since refraining from EAC is an avoidance behaviour, all messages were loss-framed (in line with Abhyankar et al., 2008; Jones et al., 2006). Ethical approval was granted from the School of Psychology ethics committee at University of Leeds (#UoL/PSY/2008_BM_01) on 1 March 2008.

### 2.2. Participants

**Figure 1:**
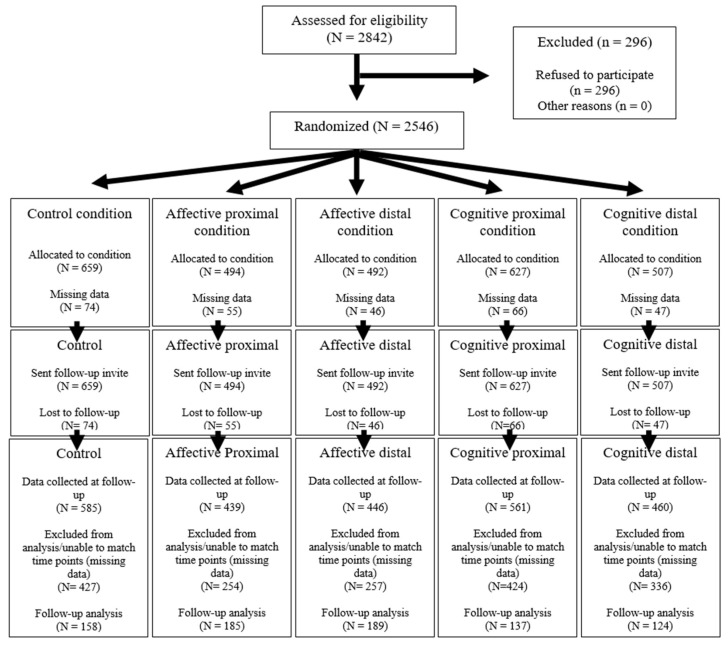
Participant flow diagram and study design.

Participants were recruited through email lists collated from university websites in the UK (totalling 42 institutions/412 departments). Students received an email inviting them to visit a website and take part in a study on health behaviour change. Where student email addresses were not available, department/school secretaries were asked to forward the request to take part in the study to their student groups. For this reason, the precise number of students initially approached is not available. A priori power calculations indicated that a minimum of 124 participants per messaging group was required to detect a small effect size (d = 0.20). This indicated that the total sample size would need to be at least 620 participants. Data collection took place over a five-week period. Figure 1 shows the participant flow diagram and study design. Simple randomisation was used by the webpage that hosted the study, which allocated participants to message condition randomly. One week later at follow-up, 2419 participants were invited to visit the website and, due to incomplete data sets (i.e., participants who had not fully completed the SRHI, gender, and NfC measures), the final number for analysis was 793 (258 men; 497 women; a ratio of 1:1.9; mean age = 23 years (SD = 6.05), with a range of 18–67 years of age). For the level of study, the sample consisted of 1st year undergraduate (N = 218), 2nd year undergraduate (N = 169), 3rd year undergraduate (N = 154), postgraduate (N = 113), PhD (N = 20), and did not specify (N = 81). The number of participants in each condition was as follows: control, N = 158; affective proximal, N = 185; affective distal, N = 189; cognitive proximal, N = 137; cognitive distal N = 124. Each at least met the minimum group sample size.

### 2.3. Measures

Habitual strength towards alcohol consumption was measured using the Self-reported Habit Index (SRHI; Verplanken & Orbell, 2003). A total of 12 items tapped the strength of habits towards EAC (e.g., “Excessive alcohol consumption is something I do frequently …”, 1 = strongly disagree to 7 = strongly agree; author-reported Cronbach’s between α = 0.89 and 0.92, present Cronbach’s at baseline α = 0.96, and follow-up α = 0.96). The SRHI has been shown to be useful to determine habit strength without measuring behavioural frequency. A high score on this scale indicates a strong habit strength. Items in the scale were orientated towards binge drinking.

NfC was measured using 18 items (e.g., “I find satisfaction in thinking hard and for long hours …”), all of which were responded to on seven-point likert scales (Cacioppo et al., 1984; Cronbach’s α = 0.78). A high score on this scale indicates a strong NfC. The median presently reported here is 4.30, previous studies have found values between 4.02 and 4.06 for NfC (Cacioppo et al., 1983).

### 2.4. Intervention

The generic message read, “Many regard drinking as part of our culture and we feel comfortable with it. Indeed, drinking in moderation does not present a major health problem. However, in recent years, there has been a growing national concern about the way that British people drink particularly in relation to the phenomenon of excessive alcohol consumption … in particular the Government is concerned about the health effects and the impact on anti-social behaviour of this type of drinking …”. In the conditions other than the control, this was accompanied by either a set of affective (affective proximal message, e.g., “episodes of excessive alcohol consumption will often lead to hangovers which create a cycle of waking up feeling ill, anxious, jittery and guilty”) or cognitive (cognitive proximal message, e.g., “excessive alcohol consumption can lead to loss of brain cells and short term memory impairment”) message information, framed in terms of the short-term (cognitive proximal message, e.g., “excessive alcohol consumption can result in 14% of individuals having unsafe sex and 10% of those unable to remember the night before. Unprotected sex is a significant contributor to unwanted pregnancies”) or long-term (cognitive distal message, e.g., “excessive alcohol consumption can result in 14% of individuals having unsafe sex. Unprotected sex is a significant contributor to contracting HIV aids and other serious sexually transmitted infections”) consequences of EAC. Despite the 7-day follow-up time frame, it was felt that these messages would target different components of ELM (Petty & Cacioppo, 1986), and as such functioned in discrete ways. These messages were piloted in a separate sample of 204 students who received one of the messages (affective or cognitive, proximally or distally salienced). Participants assessed messages on affective and cognitive strength and on perceived time valence (see Supplementary File S1 for further details of this pilot).

### 2.5. Procedure

Participants received an email invitation to take part in the study. Clicking on the hyperlink in the email invitation took them to a webpage outlining the nature of the study, with subsequent pages collecting various pieces of information. At baseline, demographic information was requested first (e.g., age, gender), followed by the baseline SRHI score, health message, and then a range of questions focusing on EAC. A definition of EAC was also provided (for both men and women).

At follow-up, participants were sent a reminder email inviting them to complete the follow-up measures. Clicking on the embedded hyperlink took them to the first page of the website. Questionnaire items measured behaviour using the same self-reported behaviour measure as used at baseline. Following this, a measure of NfC was then completed. This individual difference variable remains stable across short periods of time (certainly across a one-week period, as it was here), so it was felt that leaving this towards the end of follow-up would not be detrimental to measurement precision.

## 3. Results

Randomisation to one of the five conditions was successful in so far as there were no significant differences between the conditions by age (F(4,2778) = 0.56, *p* = 0.69), gender (χ^2^ (1, N = 4) = 10.76, *p* = 0.22), ethnicity (χ^2^ (1, N = 284) = 402, *p* = 0.13), educational level (χ^2^ (1, N = 20) = 35.99, *p* = 0.06), or amount of alcohol consumed over the preceding seven-day period (F(4,733) = 0.75, *p* = 0.56). Participants who completed both time points did not differ significantly by age (F(4,733) = 0.87, *p* = 0.48), gender (χ^2^ (1, N = 8) = 4.29, *p* = 0.37), or ethnicity (χ^2^ (1, N = 372) = 298.97, *p* = 0.26). See Table 1 for the breakdown by condition. There was also no significant difference in allocations to conditions by educational level (χ^2^ (1, N = 24) = 23.47, *p* = 0.27). See Table 2 for the allocation of participants by condition and year of study.

The principal set of analyses focused on the effects of the interventions on behaviour. ANCOVA was used to compare the message condition (five levels) on behaviour, controlling for the baseline behaviour. The effect size (Cohens d) was calculated using SPSS 27.0 and was reported alongside the main effects and interactions as a measure of magnitude of these effects.

Skewness and Kurtosis values for SRHI were satisfactory across all five conditions, respectively, based on the acceptable levels to proceed with parametric analysis detailed by Hair et al. (2022). Specifically, the values were as follows: control, g_1_.701 κ−0.661; cognitive proximal, g_1_.77 κ−0.66; cognitive distal, g_1_.48 κ−1.08; affective proximal, g_1_.65 κ−0.55; and affective distal, g_1_.76 κ−0.39.

ANCOVA was used to compare habits at follow-up, whilst controlling for baseline behaviour. The independent variables were the message condition (five levels), gender (two levels), and NfC (two levels), the latter based on a median split (low ≤ 4.3; high > 4.3). There was a significant main effect of the covariate (F(2,722) = 1462.24, *p* < 0.001, d = 0.91). There were also marginally significant main effects of the message type (F(4,722) = 2.06, *p* = 0.08) and gender (F(2,722) = 3.58, *p* = 0.06), but no main effect of NfC (F(2,722) = 0.68, *p* = 0.41).

Table 3 shows the estimated marginal means for each message condition. Post hoc comparisons (least significant difference) showed that the affective distal message produced lower levels of habit strength than any other condition, except for affective proximal. The marginally significant effect of gender reflected a higher habit strength in men compared to women.

These main effects were qualified by several significant interactions. There was a significant two-way interaction between message type and gender (F(4,722) = 3.54, *p* < 0.01, d = 0.76) and message type and NfC (F(4,722) = 5.33, *p* < 0.001, d = 0.76). Finally, there was a significant three-way interaction between message type, NfC, and gender (F(9,722) = 4.39, *p* < 0.01, d = 0.76). ANCOVA indicated a significant effect across message conditions in the low NfC group (F(4,722) = 3.64, *p* < 0.01, d = 0.99), but not in the high NfC group (F(4,722) = 1.03, *p* = 0.69). Post hoc comparisons (Games–Howell) across messages in the low NfC group showed that the affective distal message produced lower levels of habit strength towards drinking than any other condition, except for affective proximal.

Finally, Table 4 shows the estimated marginal means for each message condition split by NfC and gender. ANCOVA indicated a significant effect across message conditions in the low NfC group for men (F(4,95) = 3.13, *p* < 0.05, d = 1.00), but no significant effects for women (F(4,213) = 1.33, *p* = 0.26, d = 0.98) or for either men (F(4,156) =1.30, *p* = 0.27, d = 0.99) or women (F(4,255) = 0.22, *p* = 0.93, d = 0.27) in the high NfC group. Post hoc comparisons (Games–Howell) among the men in the low NfC group indicated that the affective distal message produced lower levels of habit strength towards drinking compared to all other conditions.

## 4. Discussion

The present study builds upon dual-process models of behaviour, which differentiate between impulsive and reflective pathways in guiding human action (Hofmann et al., 2008; Strack & Deutsch, 2004). While habitual responses often arise automatically via cue-triggered behaviours on the impulsive pathway, reasoned and reflective processing, particularly in the context of health decision-making, requires deliberate cognitive engagement. This paper places an emphasis on the reflective pathway, highlighting the importance of accurate risk appraisal, which hinges on both the availability and effective utilisation of relevant information (Fischhoff et al., 1978). Central to behaviour change is how health information is framed and delivered. Messages that engage the affective system by eliciting emotional responses or highlighting anticipated emotional consequences have been shown to exert a significant influence across various health behaviours, including alcohol consumption (Conner et al., 2020; Zack, 2006). This distinction is particularly relevant when considering the temporal framing of behavioural outcomes. Consistent with temporal discounting models (Becker & Murphy, 1988), individuals often prioritise immediate rewards over delayed consequences, a phenomenon particularly evident in addictive behaviours (Acuff et al., 2024). Consequently, affective messages may be most effective when emphasising short-term consequences, aligning with the immediate nature of emotional responses.

In line with hypothesis one, there were two messages that, when tested, acutely emerged as more effective at reducing habit strength towards EAC from baseline to follow-up; these were affective proximal and affective distal messages. Both cognitive proximal and cognitive distal messages reduced the self-reported habit strength the least. The efficacy of these messages was further shown to depend on both gender differences and NfC, and these findings are the main contribution of the current research. In a more novel use of a messaging paradigm, a combination of message type (affective, cognitive) and message salience (proximal, distal) has shown that framing information in terms of affective consequences may be more useful in changing EAC than using “traditional” cognitive-type information. Furthermore, these findings illustrate the importance of considering affective message content in terms of its proximal and distal qualities. In line with hypotheses two and three, we present findings indicating that, overall, the affective distal message appeared to work best for men low in NfC in reducing self-reported habitual EAC, whereas, for women, the affective proximal message was the most effective. In contrast to this, for both men and women high in NfC, the cognitive distal message was shown to be the most effective at reducing self-reported behaviour, in greatest contrast to the cognitive proximal message. This provisionally demonstrates that NfC plays a pivotal role in the potency of specific types of affective and cognitive health messages and supports the earlier work of Conner et al. (2011) and Haddock et al. (2008). Furthermore, the apparent differences in the effectiveness of messages to modify behaviour as a function of gender and NfC indicate an essential point that behaviour change messages might be tailored to individual differences for the best effect.

Currently, there is a limited experimental literature demonstrating specific differences in emphasising the short-term or long-term benefits of engaging in health behaviour (Ditto et al., 2006; Hall & Fong, 2003; Morris et al., 2015; Orbell et al., 2004). Nevertheless, these findings may be paired well with the often compelling nature of affective message content, which provides relevance and perceived personal applicability (Peters, 2006; Rocklage & Luttrell, 2021). This work demonstrated significant differences between messages that emphasised either the short-term or long-term consequences of refraining from EAC. These findings, if replicated, have significant practical value in terms of modifying potentially harmful drinking behaviour in the university community. Here, we identify two messages that are effective in producing reductions in EAC. Interestingly, the affective proximal and the affective distal messages were similarly effective in changing self-reported EAC. The findings illustrate the importance of considering message content both in terms of the affective versus cognitive and in terms of the proximal versus distal nature of the outcomes considered.

Finally, we propose a subtle extension to the expected utility theory, arguing that the value of time discounting can be manipulated as a function of the message content being delivered. The type and temporal framing of health messaging may significantly influence behavioural intentions via their impact on outcome expectancies (Feather, 2021; Rottenstreich & Hsee, 2001). Affective messages appear to facilitate quicker, more intuitive judgements that may enhance message efficacy in fast-paced or emotionally charged contexts. Recent public health interventions such as those that promoted COVID-19 mitigation behaviours have demonstrated how information delivery can be fashioned in such a way as to promote long-term thinking relative to short-term risks (Nan et al., 2022; Kalocsanyiova et al., 2023) and the power of tailored digital health campaigns to change behaviour (Mackert et al., 2021; de Vere Hunt & Linos, 2022).

Specifically, we identify here several factors that affect message efficacy in altering habit strength. These include affectively vs. cognitively salient information, proximal vs. distal expectancies of EAC, and the effects of gender and NfC on this relationship. The practical applications of the present findings are to highlight the need for tailoring interventions to best effect behaviour change. In the present context, the intervention can be found to have adopted several behaviour change techniques outlined by the work of Michie et al. (2013) and more recently Marques et al. (2024). Specifically, here, the messages comprised information about health consequences (5.1), the salience of consequences (5.2), anticipated regret (5.5), and information about emotional consequences (5.6).

### Limitations

There are several potential issues with the present work. Firstly, there are specific difficulties inherent in measuring behaviour using a self-report tool related to social desirability bias, particularly where the behaviour focused on is susceptible to self-presentation bias. Thus, an objective measure of behaviour may have arguably been more accurate. However, there was no evidence that this measurement inaccuracy would unequally influence the results from all message conditions, and therefore, in terms of identifying the differences between these messages, this is of less concern.

Secondly, additional measures of dispositional style may have identified further potential moderation effects of such measures as the need for affect and future time focus to investigate whether certain messages would have had a more profound effect on drinking levels for individuals who had a high or low desire for specific information types (e.g., high versus low need for affect; see Conner et al., 2011).

Thirdly, the present follow-up period was seven days. An extended follow-up period may determine the longevity of these messaging effects and may represent a useful consideration in future work assessing the role of different types of messages on behaviour. Furthermore, data collection at follow-up may have been susceptible to the briefing information provided at baseline, irrespective of the messaging conditions a participant was assigned to.

Fourthly, the attrition rate from baseline to follow-up was around 64%, and therefore it is possible that a proportion of participants withdrew their participation despite being valid targets for remaining in the study. This may have unduly influenced the profile of the final sample. However, it is clear from the AC habits of those included in the study that the intervention was able to target several problematic drinkers.

Fifth, there is an arguably large gender disparity in the final sample, with more women appearing in the final data set than men.

Finally, the measures used in the pilot study to discriminate between message types had not been previously validated. Whilst presently they were demonstrated to be distinguish in terms of their content type, it is possible that the severity of their consequences could be further counterbalanced in future work.

## 5. Conclusions

The present work has demonstrated that temporal valence plays a key role in the efficacy of different types of health promoting messages; significantly, here we contrasted affective and cognitive messages. Overall, the affective distal message was found to reduce the habit strength towards EAC more so than any other message. This contrasts with both the affective proximal and cognitive distal messages that proved less effective. These findings present an important distinction between affective and cognitive information and suggest how these different types of information may be fashioned to influence EAC habits most optimally. However, this pattern of message effectiveness appears intrinsically linked to both gender and NfC. The findings of this work can usefully inform the development of a behaviour change strategy designed to reduce the negative consequences of this behaviour and its subsequent physical and psychological impairments to health and university studies. Finally, the present work would emphasise the need for pre-registered, adequately powered randomised control trials with a longer follow-up time period and objective measurements of behaviour to progress the literature further.

## Figures and Tables

**Table 1:** The mean age, gender ratio, and level of alcohol consumption at baseline by condition.

Condition	Mean Age	Gender Ratio, Men to Women	Alcohol Consumption at Baseline
Control	22.40 (5.33)	52 vs. 104	12.73 (18.05)
Affective proximal	23.08 (5.73)	61 vs. 124	12.49 (16.71)
Affective distal	22.62 (5.47)	41 vs. 90	13.67 (19.56)
Cognitive proximal	22.94 (5.66)	44 vs. 94	10.70 (14.34)
Cognitive distal	23.67 (8.05)	52 vs. 72	14.25 (22.09)

**Table 2:** The allocation of participants by condition and year of study.

Condition	Undergraduate Level			Postgraduate		Other	Not Reported
	1	2	3	M	PhD		
Control	50	38	27	15	9	1	15
Affective proximal	53	40	38	23	15	1	15
Affective distal	34	30	28	17	9	0	13
Cognitive proximal	34	32	31	19	10	0	11
Cognitive distal	37	25	25	17	12	0	8

NB randomisation to condition did not significantly differ by academic year of study (F(4,669) = 0.69, *p* = 0.60).

**Table 3:** Estimated marginal means and standard errors for self-reported habits towards EAC by message type.

Message	Estimated Marginal Mean	Standard Error	95% Confidence Interval	
Control	2.76 _d_	0.09	2.58	2.94
Cognitive proximal	2.84 _cd_	0.09	2.66	3.02
Cognitive distal	2.81 _bcd_	0.09	2.62	3.00
Affective proximal	2.72 _abcd_	0.09	2.55	2.89
Affective distal	2.47 _a_	0.11	2.27	2.68

Note: Means in a column that do not share the same postscript were significantly different based on Games–Howell post hoc test.

**Table 4:** Estimated marginal means and standard errors for self-reported habits towards EAC by message type, NfC, and gender.

NfC	Gender	Message	Estimated Marginal Mean	Standard Error	95% Confidence Interval	
Low	Men	Control	2.73 _b_	0.31	2.13	3.34
		Cognitive proximal	2.87 _b_	0.29	2.28	3.46
b < a		Cognitive distal	3.02 _b_	0.32	2.39	3.65
		Affective proximal	2.61 _b_	0.30	2.01	3.20
		Affective distal	1.30 _a_	0.42	0.48	2.12
	Women	Control	2.96 _ab_	0.18	2.60	3.32
		Cognitive proximal	3.18 _a_	0.19	2.82	3.55
		Cognitive distal	2.90 _ab_	0.19	2.45	3.07
		Affective proximal	2.63 _b_	0.17	2.30	2.97
		Affective distal	3.04 _ab_	0.18	2.69	3.39
High	Men	Control	2.83 _ab_	0.15	2.52	3.13
		Cognitive proximal	2.49 _a_	0.18	2.14	2.85
		Cognitive distal	2.76 _ab_	0.16	2.45	3.07
		Affective proximal	2.97 _b_	0.15	2.68	3.26
		Affective distal	2.95 _ab_	0.17	2.63	3.28
	Women	Control	2.65 _a_	0.10	2.44	2.85
		Cognitive proximal	2.73 _a_	0.11	2.51	2.94
		Cognitive distal	2.58 _a_	0.14	2.30	2.85
		Affective proximal	2.70 _a_	0.09	2.51	2.89
		Affective distal	2.70 _a_	0.11	2.47	2.92

Note: Means in a column that do not share the same postscript were significantly different based on Games–Howell post hoc tests.

## Data Availability

The data that support the findings of this study are available from the corresponding author, B.M., upon reasonable request.

## Supplementary Materials

The following supporting information can be downloaded at https://www.mdpi.com/article/10.3390/bs15121637/s1.
