# Supplementary material for: Changing University Students’ Habit Strength Towards Alcohol Consumption Using Affectively and Cognitively Framed Messages
Source: Behav Sci (Basel). 2025 Nov 28;15(12):1637. doi: 10.3390/bs15121637 (PMC12730057; doi:10.3390/bs15121637)
Supplement: Supplementary file 1 [file behavsci-15-01637-s001.zip › behavsci-3899318-supplementary.pdf]

Table S1

Estimated marginal means and standard error for affective, cognitive, proximal and distal ratings of all message types

	Message ratings							
	Affective		Cognitive		Proximal		Distal	
Message	Mean	SE	Mean	SE	Mean	SE	Mean	SE
Cognitive proximal	5.85	.48	9.78	.41	10.17	.39	6.41	.43
Cognitive distal	6.61	.54	9.15	.46	6.40	.45	10.09	.49
Affective proximal	8.27	.51	5.54	.43	9.74	.42	6.01	.46
Affective distal	8.73	.49	7.41	.42	7.34	.40	10.10	.44

Note: There was no significant main effect of message
